# An Inflammation-Associated Prognosis Model for Hepatocellular Carcinoma Based on Adenylate Uridylate- (AU-) Rich Element Genes

**DOI:** 10.1155/2023/2613492

**Published:** 2023-05-02

**Authors:** Li Song, Xiangzheng Su, Yao Lu, Dongliang Hua, Ziren Gao

**Affiliations:** ^1^Academy of Advanced Interdisciplinary Studies, Qilu University of Technology (Shandong Academy of Sciences), Jinan 250353, China; ^2^Department of Tissue Repair and Regeneration, The First Medical Center of Chinese PLA General Hospital, Beijing 100853, China; ^3^Xiyuan Hospital, China Academy of Chinese Medical Sciences, Beijing 100091, China

## Abstract

Hepatocellular carcinoma (HCC) is a typical inflammation-driven cancer and ranks sixth in the incidence rate worldwide. The role of adenylate uridylate- (AU-) rich element genes (AREGs) in HCC remains unclear. HCC-related datasets were acquired from The Cancer Genome Atlas (TCGA) database and Gene Expression Omnibus (GEO) database. Differentially expressed AREGs (DE-AREGs) between HCC samples and healthy controls were identified. The univariate Cox and LASSO analyses were performed to determine the prognostic genes. Furthermore, a signature and corresponding nomogram were configured for the clinical prediction of HCC. The potential signature-related biological significance was explored using functional and pathway enrichment analysis. Additionally, immune infiltration analysis was also performed. Finally, the expression of prognostic genes was verified using real-time quantitative polymerase chain reaction (RT-qPCR). A total of 189 DE-AREGs between normal and HCC samples were identified, wherein CENPA, TXNRD1, RABIF, UGT2B15, and SERPINE1 were selected to generate an AREG-related signature. Moreover, the prognostic accuracy of the AREG-related signature was also confirmed. Functional analysis indicated that the high-risk score was related to various functions and pathways. Inflammation and immune-related analyses indicated that the difference of T cell and B cell receptor abundance, microvascular endothelial cells (MVE), lymphatic endothelial cells (lye), pericytes, stromal cells, and the six immune checkpoints was statistically significant between the different risk groups. Similarly, RT-qPCR outcomes of these signature genes were also significant. In conclusion, an inflammation-associated signature based on five DE-AREGs was constructed, which could act as a prognostic indicator of patients with HCC.

## 1. Introduction

Hepatocellular carcinoma (HCC) is one of the most common cancers and has the second-highest mortality rates worldwide [1]. Currently, the standard treatment for HCC includes resection, local therapies such as ablation and radiotherapy, and liver transplantation. However, owing to the high recurrence and mortality rates, the prognosis of patients with HCC remains unsatisfactory [2, 3].

As an essential cis-acting short sequence in the 3′UTR, adenylate uridylate- (AU-) rich element (ARE) has a significant effect on mRNA stability and translation and is closely related to mRNA decay [4, 5]. Chen and Shyu reported three classes (class I, class II, and class III) of ARE, which were based on the presence of an AUUUA motif in the U-rich region [6]. Specifically, among U- or AU-rich sequences and repeated sequences of AUUUA or non-AUUU overlapping pentamers determined as ARE sequences, the latter two forms are considered to be the least functional ARE sequences [4, 7]. Moreover, the AU-rich binding factor 1 (AUF1) is a well-known ARE-specific RNA-binding protein (ARE-BPs). Zhang et al. reported a novel role of AUF1 in promoting the development and drug resistance of HCC [8] Furthermore, if the degradation of ARE-mRNAs was destroyed, chronic inflammation will be induced. For example, IL-17, a mediator implicated in chronic and severe inflammatory diseases, can enhance the production of proinflammatory mediators by attenuating the decay of ARE-mRNAs [9]. Similarly, the correlation of the biomarkers relevant to inflammatory response disorder and HCC prognosis was explored by Xing et al., wherein an inflammation-related gene (IRG) risk model comprising six IRGs that could identify tumors with low immune levels and also indicate the efficacy of immunotherapy was constructed [10].

HCC is a typical inflammation-driven carcinoma with progressive chronic nonresolving inflammation [11]. In addition, owing to the disruption of the degradation progress of ARE-mRNA, chronic inflammation and cancer are considered potential outcomes [12]. For example, the ARE gene uPA is upregulated in various cancers and stimulates angiogenesis, providing tumor cells with abundant nutrition and oxygen [13]. COX-2 in ARE genes contributes to angiogenesis, metastasis, and other tumor-related mechanisms in colon cancer [14]. Additionally, AUBPs contain typical sequences that are rich in AU bases (AREs) and can rapidly regulate 3′-UTR harbouring ARE-binding motifs of liver disease-related cytokines and proinflammatory molecules. AUBPs could also be considered effective factors in HCC progression [15]. Therefore, ARE genes are speculated to be closely related to tumors and have promising potential prognostic value as a new target for tumor therapy.

This study is aimed at demonstrating the prognostic value of the ARE genes for the first time in HCC using bioinformatics analysis and exploring its potential therapeutic agents. This study is also aimed at aiding in the theoretical guidance for the treatment of HCC.

## 2. Materials and Methods

### 2.1. Data Source

The TCGA-LIHC datasets were downloaded from The Cancer Genome Atlas (TCGA) database (https://portal.gdc.cancer.gov/), including 374 samples with HCC and 50 controls. A total of 421 non-formalin-soaked tumor tissues and normal tissues (50 normal samples, 371 HCC samples) were selected for differential expression analysis. Moreover, 363 samples were retained for constructing a prognostic model based on extracted complete survival status and clinical information. The GSE14520 dataset was downloaded from the Gene Expression Omnibus (GEO) database (https://www.ncbi.nlm.nih.gov/gds/) for external validation, wherein 221 case samples had complete survival information. Furthermore, the GSE54236 dataset containing 81 HCC tissue samples and 80 adjacent nontumor samples was utilized for gene expression analysis. Additionally, 4884 AREGs were downloaded from the Adenylate Uridylate-Rich Element Database (ARED, https://brp.kfshrc.edu.sa/ared).

### 2.2. Differential Expression Analysis

Limma within R was applied to select differentially expressed genes (DEGs) (*p* < 0.05 and |Log2FC| > 1) between the HCC and healthy groups in the TCGA-LIHC datasets [16]. Following this, TBtools were used to intersect DEGs and ARE genes to obtain differentially expressed AREGs (DE-AREGs) [17].

### 2.3. Functional and Pathway Enrichment Analysis of DE-AREGs

The Gene Ontology (GO) and the Kyoto Encyclopedia of Genes and Genomes (KEGG) enrichment scores of DE-AREGs were further analysed using “clusterProfiler,” with *p*.adj < 0.05 and *q* value cutoff = 0.2 determining statistical significance [18].

### 2.4. Construction and Validation of the DE-AREG Signature

A total of 363 HCC samples with complete survival and clinical information were used as the training set to construct an AREG-related signature. Moreover, prognostic DE-AREGs were screened using univariate Cox and LASSO regression methods, which were performed using the survival and glmnet package, respectively [19, 20]. Subsequently, the multivariate Cox regression was used to construct the DE-AREG signature. The risk score was calculated as follows: Risk score = *h*0(t) ∗ exp (*β*1*X*1 + *β*2*X*2 +⋯+ *βnXn*). Following this, the training set was classified into high- and low-risk groups based on the median value among risk scores of patients of HCC. Kaplan-Meier (K-M) curves were plotted using the survminer package [21]. The receiver operating characteristic (ROC) curve and the area under the curve (AUC) were drawn using the R package survival ROC [22]. Finally, the GSE14520 dataset was used to validate the prognostic performance of the signature.

### 2.5. Independent Prognostic Analysis

The wilcox.test function in R was used to evaluate the clinical relevance of the risk model based on the clinical data of HCC samples. Using the univariate and multivariate Cox regression models, the independent prognostic factors and relevant clinical parameters (*p*-value < 0.05) were used to establish the prognostic nomogram.

### 2.6. Functional Enrichment Analysis

To further investigate the functions related to the DE-AREG signature, gene set enrichment analysis (GSEA) was conducted using the gene expression data extracted from the two risk groups. c5.go.v7.4.symbols.gmt (GO) and c2.cp.kegg.v7.4.symbols.gmt (KEGG) were selected as reference gene sets. The pathways and GO terms with |NES| > 1, NOM *p* value < 0.05, and FDR *q*value < 0.25 were extracted for further analysis.

### 2.7. Inflammatory and Immune-Related Analyses

The differences in inflammation-related factors, cytolytic score (CYT), antigen presentation mechanism (APM), infiltration of vascular cells, and immune checkpoints were interpreted in the two risk groups using xCell [23]. Additionally, the single sample GSEA (ssGSEA) algorithm was used to estimate the relative abundance of 28 immune cells for comparison between the risk groups [19]. Correlation analysis was performed to identify the relationship between immune cells and risk score. Next, overlapping immune cells with *r* > 0.3 were obtained that could be associated with different risk groups.

### 2.8. Prediction of Potential Biomarker-Drug Interactions

The potential drugs for the signature genes were predicted based on the Binding DB database (https://www.bindingdb.org/bind/index.jsp), STRING database (https://cn.string-db.org), and ZINC15 database (https://zinc15.docking.org/). In the Binding DB database, the drugs with affinity value < 50 were selected first, and then, these drugs were screened in the STRING database with a confidence value = 0.85. In the ZINC15 database, the potential drugs with the lowest affinity according to the molecular docking score were selected.

### 2.9. Real-Time Quantitative Polymerase Chain Reaction (RT-qPCR) Analysis and Validation of the Signature Genes in Cell Lines

Total RNA was collected from the nontumorigenic hepatocyte cell line (WRL68) and three HCC tumor cell lines (Huh-7, HepG2, and Sk-Hep-1) using a TRIzol reagent (Invitrogen, Eugene, OR, USA). The first-strand cDNA was synthesized with superScript RT I First-Strand cDNA Synthesis All-in-One™ First-Strand cDNA Synthesis Kit (Servicebio, Wuhan, China). The 2x Universal Blue Sybr Green qPCR Master Mix (Servicebio, Wuhan, China) was used for RT-qPCR detection. The primers used in this study are presented in Table 1. The 2^-*ΔΔ*Ct^ method was used for the expression detection of the signature genes [24].

## 3. Results

**Figure 1:**
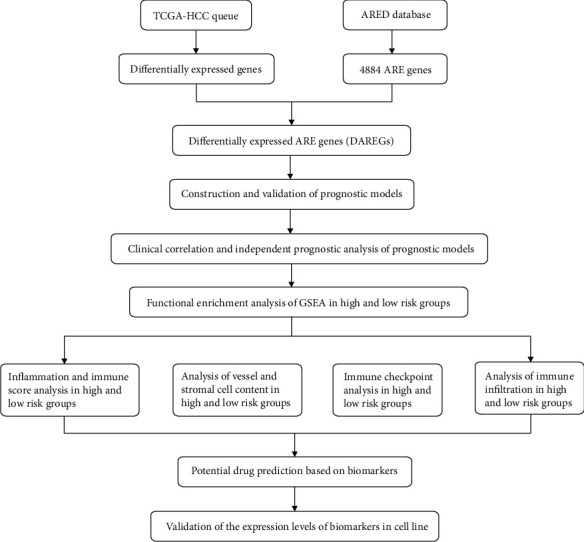
Workflow of the study on AREG prognostic signatures for HCC.

We conducted our study as presented in the workflow (Figure 1). A total of 1512 genes were identified as differently expressed at mRNA level in tumor tissues (*n* = 371) when compared with that of normal tissues (*n* = 50) [25]. Moreover, the following software was used in this study: xCell (v.1.1.0), limma (v.3.44.3), ggplot2 (v.3.3.2), TBtools (v.1.098661), clusterProfiler (v.3.16.0), Survival (v.3.2 3), pROC (v.1.16.2), psych (v.2.0.9), GSVA (v.1.38.2), rms (v.5.4-1), and Vina (v.1.1.2).

### 3.1. Identification of DE-AREGs

**Figure 2:**
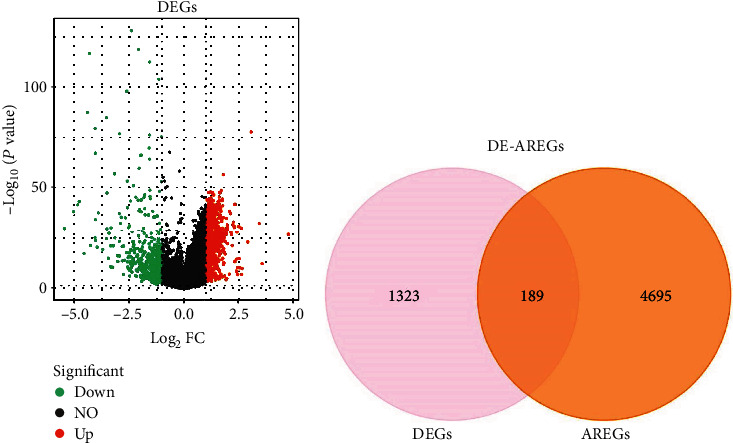
A total of 180 DE-AREGs were selected. (a) Volcano plot of 1512 differentially expressed genes (DEGs). Red, upregulation; green, downregulation. (b) Venn diagram of the 189 overlapping genes.

We identified 1512 DEGs between HCC and normal samples, including 1046 upregulated and 366 downregulated DEGs (Figure 2(a)). Following this, 189 overlapping genes were obtained between 1512 DEGs and 4884 AREGs, which were considered DE-AREGs (Figure 2(b)). Furthermore, the hypergeometric distribution of the intersection data in the Venn graph was analysed using the Phyper function of R language. A significant overlap between the non-DEGs and AREG sets was observed (*p* = 2.42*e* − 46), indicating that the AREGs tend to be stable between tumor and normal samples.

### 3.2. Functional and Pathway Enrichment of Different Risk Groups

**Figure 3:**
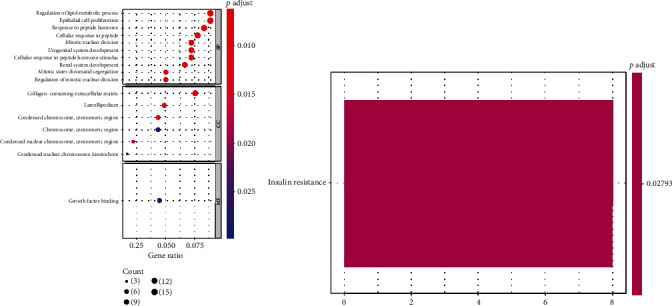
Functional enrichment analysis. (a) GO annotation of DE-AREGs with the top 10 enrichment scores. (b) Top 10 KEGG pathways of DE-AREGs.

From the perspective of the biological roles of the 189 DE-AREGs, a total of 59 GO terms were enriched, including biological process (BP) of 52 terms, cell component (CC) of six terms, and one molecular function (MF) term. GO BP analysis suggested that 189 DE-AREGs were relevant to mitotic nuclear division, regulation of lipid metabolic process, epithelial cell proliferation, etc. (Figure 3(a)). For GO CC analysis, the top three enriched terms were condensed chromosome, centromeric region, and collagen-containing extracellular matrix. For GO MF analysis, 189 DE-AREGs were related to growth factor binding. Furthermore, the KEGG pathway showed enrichment in the insulin resistance pathway (Figure 3(b)).

Furthermore, enrichment analyses of the aforementioned DE-AREGs with different expression trends indicated that the upregulated genes enriched 19 GO terms and downregulated genes enriched two GO terms; however, no KEGG pathway enrichment was observed. Moreover, the downregulated genes annotated in GO terms included core promoter sequence−specific DNA binding and neurotrophin receptor binding of the MF category. The upregulated genes mainly enriched in GO terms included epithelial cell proliferation, regulation of lipid metabolic process, response to peptide hormone, urogenital system development, and renal system of BP (Figure S1).

### 3.3. Construction of an AREG-Related Signature

**Figure 4:**
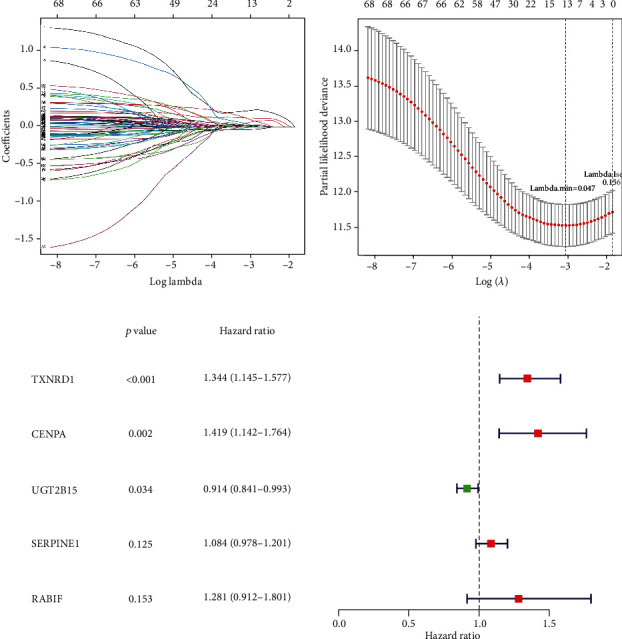
Five signature genes were generated based on the LASSO Cox regression. (a, b) Construction of the LASSO Cox regression model. (c) Forest map of the multivariate Cox results.

The univariate Cox regression analysis was first performed with 189 DE-AREGs, and 72 DE-AREGs were screened (Table S1). Subsequently, the LASSO Cox analysis on the 72 DE-AREGs showed that 13 DE-AREGs were potential prognostic genes at the lambda.min = 0.047 (Figures 4(a) and 4(b)). Moreover, the adopted multivariate Cox analysis identified five 5 signature genes (CENPA, TXNRD1, RABIF, UGT2B15, and SERPINE1) (Table 2 and Figure 4(c)). Furthermore, the five DE-AREG expressions were validated in the GSE14520 and TCGA datasets. The expression levels of *CENPA*, *TXNRD1*, and *RABIF* in the HCC groups were significantly higher than that in the normal groups. Contrarily, *UGT2B15* and *SERPINE1* were significantly higher in the normal groups compared with the HCC samples (Figure S2).

### 3.4. Evaluation and Validation of the AREG-Related Signature

**Figure 5:**
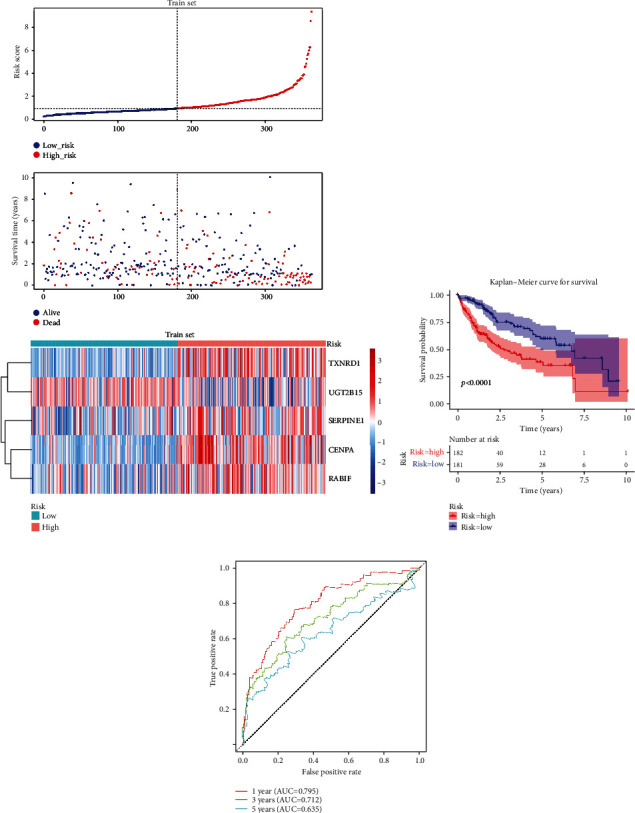
The prognostic value of the five gene signatures in the training set. (a) Distribution of risk score, survival time, and heatmap of the five gene signatures in the training set. (b) Kaplan-Meier curve of patients with HCC having different risk scores (*p* < 0.0001). (c) The receiver operating characteristic (ROC) curve evaluating the validity of the risk model.

Risk score = 0.3501952 × CENPA + 0.295307 × TXNRD1 + 0.2479362 × RABIF + (−0.0899647) × UGT2B15 + 0.0804323 × SERPINE1. The samples of the training set were divided into the high- (182 HCC samples) and low-risk (181 HCC samples) groups (median risk score = 0.9064) (Figure 5(a)). Additionally, the overall survival (OS) of HCC samples showed that a higher risk score was accompanied by poorer OS (Figure 5(b)). Furthermore, the ROC curve revealed that the AUC was greater than 0.6 (Figure 5(c)).

**Figure 6:**
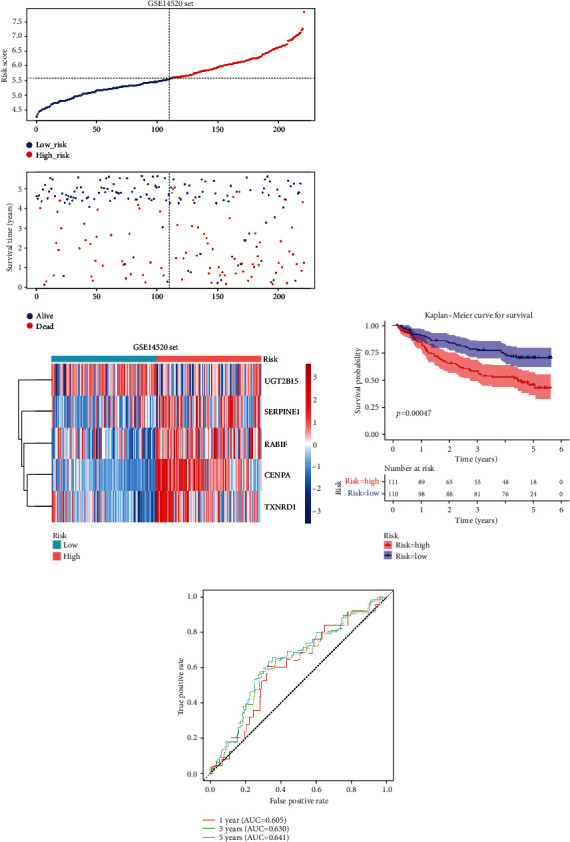
Risk model validation is in the GSE14520 dataset. (a) Risk score, survival time, and gene expression heatmaps were plotted in the validation set (GSE14520 dataset). (b) Kaplan-Meier analysis in the validation set (GSE14520 dataset) (*p* < 0.001). (c) The receiver operating characteristic (ROC) curve evaluating the validity of the risk model.

Furthermore, the five DE-AREG prognostic signatures were verified in the GSE14520 dataset. The GSE14520 dataset was also divided into two risk groups (cutoff = 5.581) (Figure 6(a)). The performance of the K-M analysis and AUC values were per the training set (Figures 6(b) and 6(c)), indicating that the five AREG-related signatures had higher specificity and sensitivity for HCC.

### 3.5. Independent Prognostic Value of the DE-AREG Prognostic Signature and Construction of a Predictive Nomogram

**Figure 7:**
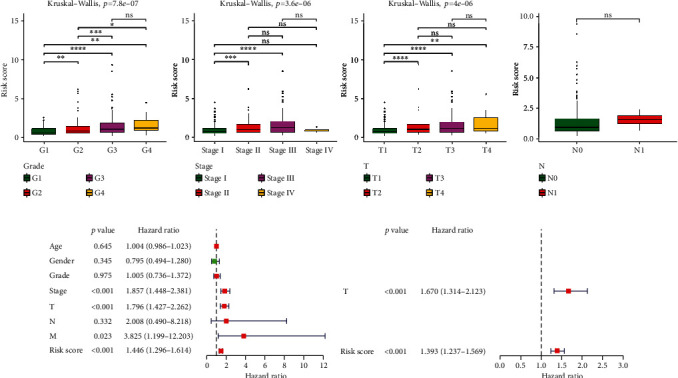
Correlation analysis between AREG-related signature and clinicopathological features. (a) Correlation of risk score and clinicopathological features. The abscissa represents clinical traits, and the ordinate represents risk score. ^∗^*p* < 0.05, ^∗∗^*p* < 0.01, ^∗∗∗^*p* < 0.001, and ^∗∗∗∗^*p* < 0.0001. ns: not significant. (b) Forest plots of independent prognostic-univariate Cox results. (c) Forest plots of independent prognostic-multivariate Cox results.

To estimate the correlations between the AREG-related signature and clinicopathological features, the risk score and clinicopathological information were combined. As shown in Figure 7(a), the grade, stage, and T stage were significantly different. Univariate analysis suggested that stage, T/M stage, and risk score were considered essential to the prognosis of HCC (*p* < 0.05) (Table 3 and Figure 7(b)). Additionally, the T stage and risk score had an independent prognostic value for HCC (*p* < 0.05) (Table 4 and Figure 7(c)).

**Figure 8:**
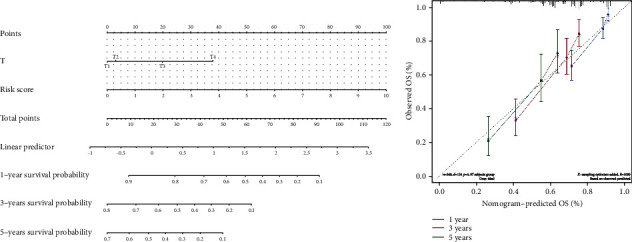
Evaluation of the clinical benefit of the risk score. (a) The nomogram to predict the survival rate of patients with hepatocellular carcinoma (HCC). (b) Calibration curves of the nomogram.

Furthermore, a nomogram was generated with T stage and risk score as the variables (Figure 8(a)), and the C-index of 1, 3, and 5 years indicated that the nomogram performed well (Figure 8(b)).

### 3.6. GO and KEGG Pathways Enriched in the Two Risk Groups

**Figure 9:**
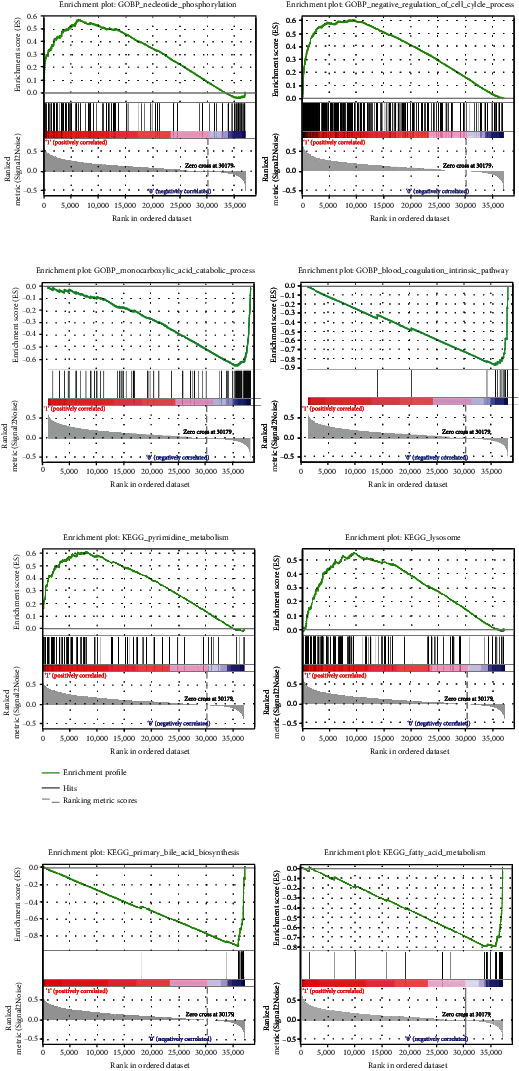
Gene set enrichment analysis outcomes in different risk groups. (a) The Top2 GO significant enrichment in the high-risk group. (b) The Top2 GO significant enrichment in the low-risk group. (c) The Top2 KEGG significant enrichment in the high-risk group. (d) The Top2 KEGG significant enrichment in the low-risk group.

To explore the biological function of the DEGs between different risk groups, functional and pathway enrichment was performed. A total of 2242 GO annotations were correlated with high-risk scores (Table S2 and Figure 9(a)), such as nucleotide phosphorylation and negative regulation of the cell cycle process. The low-risk group was found to affect the monocarboxylic acid catabolic process and blood coagulation intrinsic pathway (Table S3 and Figure 9(b)). Moreover, 72 KEGG pathways were enriched in the high-risk group, such as pyrimidine metabolism, cell cycle, and lysosome (Table S4 and Figure 9(c)). A total of 13 KEGG pathways were enriched in the low-risk group, such as primary bile acid biosynthesis, fatty acid metabolism, and retinol metabolism (Table S5 and Figure 9(d)).

### 3.7. Difference Analyses of Inflammation, Immune, Vascular Cells, and Stromal Cells with Different Risk Scores

**Figure 10:**
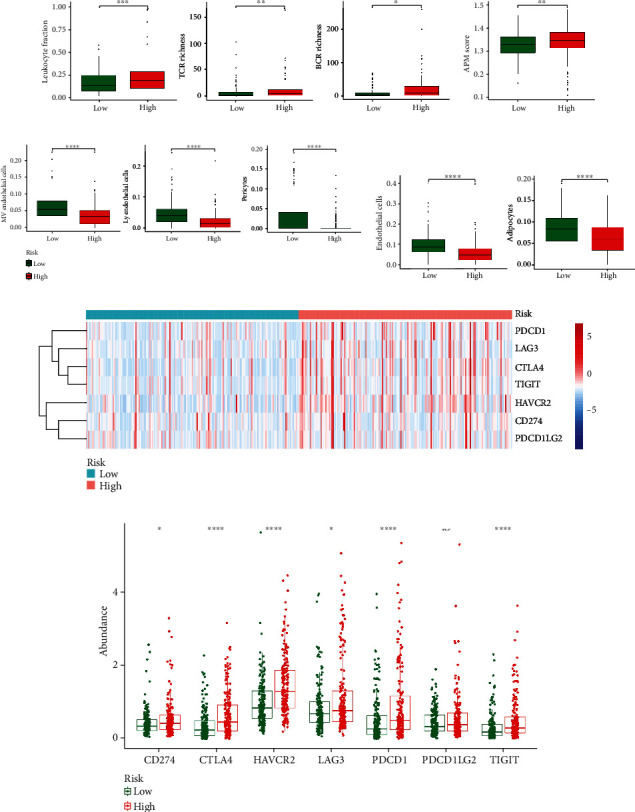
Analysis of inflammation and immune response. Differences in inflammatory immune factors (a), APM score (b), vascular cell infiltration (c), and stromal cell infiltration (d) in different risk groups were displayed. (e, f) Heatmap and box plots of immune checkpoint expressions in different risk groups. ^∗^*p* < 0.05, ^∗∗^*p* < 0.01, ^∗∗∗^*p* < 0.001, and ^∗∗∗∗^*p* < 0.0001. ns: not significant.

Inflammation and immune infiltration were validated to be critical to HCC development. Additionally, T cell and B cell receptor abundance and APM levels were significantly upregulated in the low-risk group (Figures 10(a) and 10(b)). Moreover, in the low-risk group, higher cell infiltrations were observed in microvascular endothelial cells (MVE), lymphatic endothelial cells (lye), pericytes, and stromal cells (Figures 10(c) and 10(d)). The immune checkpoints of PDCD1, CTLA4, HAVCR2, and TIGIT had a significant difference in different risk groups (Figures 10(e) and 10(f)).

**Figure 11:**
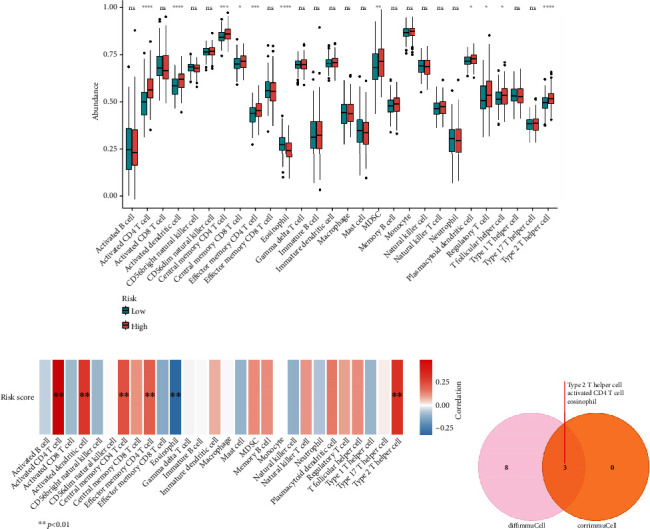
Immunoinfiltration analysis. (a) Box plot of the 28 immune cell differences in the two risk groups. (b) Heatmap of immune cell correlation with a risk score. (c) Venn diagram of 11 differentially expressed immune cell types and three immune cell types significantly correlated with risk score (*r* > 0.3). ^∗^*p* < 0.05, ^∗∗^*p* < 0.01, ^∗∗∗^*p* < 0.001, and ^∗∗∗∗^*p* < 0.0001. ns: not significant.

Furthermore, the results of immune cell infiltration suggested that 11 immune cells were significantly different such as activated CD4 T cells and eosinophils (Figure 11(a)). Moreover, five cell types, such as activated CD4 T cell and type 2 T helper cell, were positively correlated with risk scores (Figure 11(b)). However, there was a significantly negative correlation between eosinophil and risk score (Figure 11(b)). Finally, activated CD4 T cells, type 2 T helper cells, and eosinophils were identified as key immune cells in different risk groups (Figure 11(c)). Additionally, the hypergeometric distribution of the intersection data in the Venn graph was validated using the Phyper function of R language (*p* = 0.05).

### 3.8. Potential Drug Prediction

**Figure 12:**
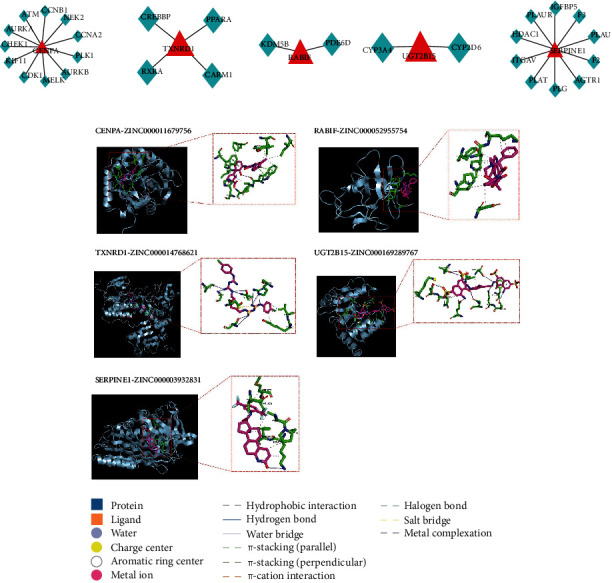
Prediction for the potential drugs of biomarkers. (a) Potential drug targets for biomarkers. Red triangles represent biomarkers and green diamonds represent drug targets. (b) Molecular docking complex and element legend.

To investigate the potential drugs that regulate signature genes, the predictions were performed based on the Binding DB database and ZINC15 database. In the Binding DB database, 11 drug targets were predicted for CENPA; four drug targets were predicted for TXNRD1; two drug targets were predicted for RABIF; two drug targets were predicted for UGT2B15; 10 drug targets were predicted for SERPIN1 (Figure 12(a)). In the ZINC15 database, the target drugs of TXNRD1, CENPA, UGT2B15, SERPINE1, and RABIF were ZINC00014768621, ZINC000167289767, ZINC000003932831, and ZINC000052955754, respectively (Figure 12(b)).

### 3.9. Validation of Signature Genes in Cell Lines by Using RT-qPCR

**Figure 13:**
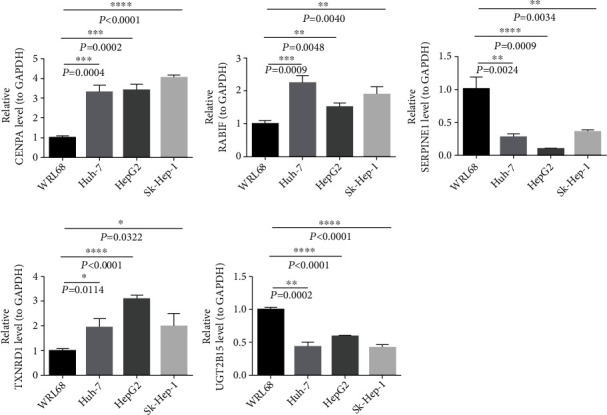
Real-time quantitative polymerase chain reaction (RT-qPCR) validation of five signature genes in hepatocellular carcinoma cells and controls.

Differences in the expression of the five signature genes between the nontumorigenic hepatocyte cell line (WRL68) and three HCC tumor cell lines (Huh-7, HepG2, and Sk-Hep-1) were compared using RT-qPCR (Figure 13). *UGT2B15* and *SERPINE1* were significantly higher in WRL68 than in HCC cells. However, the mRNA levels of RABIF, CENPA, and TXNRD1 were lower in WRL68 compared with HCC cells.

## 4. Discussion

Owing to the progressivity of HCC, it is necessary to establish reliable prognostic signatures for HCC diagnosis and treatment. Computational models have recently become an effective adjunct to explore possible carcinogenic factors and biomarkers for HCC [26]. Additionally, several vital proteins were identified that were coded by AU-rich mRNAs, which play a similar role in inflammation and cancer development [4].

For the treatment of HCC, histological grades and gene expression data were utilized to construct a novel signature for the prediction of HCC prognosis [27]. Moreover, immune-related genes and corresponding potential compounds were investigated in HCC [24]. In this study, a five DE-AREG-based prognostic signature was generated and validated. Furthermore, several potential drugs were identified, providing a reference for HCC treatment. Additionally, RT-qPCR results confirmed the expression of the prognostic genes in HCC cell lines.

RNA-seq data in the TCGA-HCC datasets was conducted to investigate biomarkers related to HCC prognosis, wherein a prognostic model comprising CENPA, TXNRD1, RABIF, UGT2B15, and SERPINE1 was constructed. Functional and pathway enrichment analyses of these model genes showed that they could play an important role in the prognosis of patients with HCC using various pathways. Specifically, CENPA (centromere protein A), an essential factor in cell division, acts on centromeres and kinetochores. A study on breast cancer indicated that the functional alteration of the CENPA-related coexpression network can affect and contribute to the development of various cancers by targeting the process of cell cycle progression [28]. Additionally, a study related to HCC prognosis revealed that high expression levels of CENPA were correlated to poor prognosis in patients with HCC [29], which was consistent with this study's findings where CENPA was considered an unfavourable prognostic factor for HCC prognosis. Furthermore, in the current study, the cell cycle-related KEGG pathway was significantly enriched in the high-risk group. The expression of CENPA was also observed to be lower in HCC samples compared to normal samples, indicating that CENPA could play an important role in the prognosis of HCC patients via cell cycle-related pathways. The overexpression of *TXNRD1* (thioredoxin reductase 1) had been reported as a promising therapeutic factor in HCC [30]. Conversely, the lipid peroxidation-related gene *SLC27A5* was found to downregulate TXNRD1 expression and inhibit HCC progression [31]. Notably, the fatty acid metabolism pathway was activated in the low-risk group, indicating that the imbalance between TXNRD1 mRNA expression and fatty acid metabolism regulation could promote poor prognosis in patients with HCC. Furthermore, *RABIF* (RAB interacting factor) was mutated in GTPase Sec4 and was speculated to be involved in cancer cell progression, invasion, and metastasis [32, 33]. The RT-qPCR results also demonstrated the increased expression of RABIF in HCC cell lines. Uridine diphosphate glucuronic acid transferase (UGT) is a crucial phase II metabolism enzyme in the human body, mainly found in liver microsomes. Moreover, studies have demonstrated that the homozygous D85 UGT2B15 (UDP glucuronosyltransferase family 2 member B15) allele genotype could be associated with an increased risk of prostate cancer [34]. In this study, the univariate Cox analysis revealed that UGT2B15 was a favourable prognostic factor. The RT-qPCR analysis also revealed that UGT2B15 mRNA was lower in the three HCC cell lines than that in the control group, indicating the favourable prognostic value of UGT2B15 in HCC. SERPINE1 (serpin family E member 1) could promote the malignant transformation of chronic hepatitis to HCC by targeting miR-145 [35–37]. Hachim et al. indicated that SERPINE1 is also closely associated with the cell cycle process [38]. Consistently, multivariate Cox results suggested that SERPINE1 was an unfavourable prognostic factor for HCC. Notably, the gene expression results showed that SERPINE1 was expressed lower in HCC samples than in paracancerous tissues, which was contradictory to the multivariate Cox results. Thus, we hypothesised that this phenomenon could be due to the complex mechanism of genes and disease; however, further experimental verification is needed.

Next, we analysed inflammatory and immune-related differences between the risk subgroups associated with the five DE-AREG prognostic models. The results indicated an inconsistent immune microenvironment and inflammatory status between the two risk subgroups. Through ssGSEA analysis of the three essential immune cells, it was revealed that activated CD4 T cell, type 2 T helper cell, and eosinophil have a great relationship with the five DE-AREG prognostic models. First, activated CD4 T cells in HCC could induce the generation of IgG-producing plasma cells with the assistance of macrophages. IgG further inhibited the tumor immune response by producing cytokines [39]. Second, the neddylation pathway was activated in HCC and changed with disease development. Herein, we revealed that an activated neddylation pathway was accompanied by a higher infiltration of Th2 cells. Meanwhile, the immunosuppressive effects of IL-4 and IL-10 secreted by Th2 cells could further regulate tumor growth and metastasis [40, 41]. However, Th2-released cytokines were also influenced by the Th1/Th2 imbalance in patients with HCC [42, 43]. Additionally, it was reported that eosinophils, originally located in the primary cancer cells, could be stimulated by eosinophilic chemokines and transported into the liver to promote cancer development [44, 45]. These findings provided more possibilities by targeting immunotherapy for HCC treatment.

Cui et al. identified five prognosis-related metabolic genes that were involved in the dysregulation of the metabolic microenvironment in the survival prognosis model of patients with HCC, which was constructed using TCGA-LIHC. They also reported on the use of these genes in metabolic therapy [46]. Moreover, based on TCGA-LIHC and GSE14520 datasets, the prognosis model of HCC, which included a nine-gene amino acid metabolism-related risk signature, represented only amino acid metabolisms that cause liver cancer. Meanwhile, amino acid metabolism therapy was also proposed [47]. Shen et al. constructed a ten-immune-related gene risk model to predict the survival of patients with HCC in terms of immune regulation, providing a novel target for the treatment of patients with HCC [48]. In comparison, the five DE-AREG prognostic models in this study present more carcinogenic factors. Additionally, multiangle therapy could be used to guide clinical therapy, such as using multiple types of anticancer drugs based on the five signature gene targets. Moreover, this model could improve the treatment options for patients with HCC.

Currently, the presence of cirrhosis causes a considerable challenge to the surgical treatment of HCC [1, 24]. Liver transplantation has many limitations, such as the lack of an appropriate liver source or graft rejection [24, 49]. In the treatment of patients with advanced HCC, such as first-line sorafenib and second-line regorafenib, only certain patients exhibited good liver function [50, 51]. Therefore, bioinformatics analysis based on next-generation sequencing is becoming an important method to identify biomarkers and explore therapeutic drugs and pathogenesis. Moreover, ARE genes can reliably predict the OS of patients with HCC, and the prognostic signature was relevant to the inflammation-associated element. However, to provide patients with a better prognosis and aid in personalised targeted therapy, further prospective trials to test the clinical efficacy of the signature should be conducted.

## 5. Conclusions

Using three cohort profile datasets and integrated bioinformatics analysis, five DE-AREGs were identified and referred to as biomarkers of the inflammation-associated prognostic model in HCC. The novel DE-AREG-based risk scoring system was established for the clinical assessment of patients with HCC.

## Figures and Tables

**Table 1:** Primers for real-time quantitative polymerase chain reaction (RT-qPCR).

Primer		Sequence
UGT2B15	F	ATTTCTGTTCCCTCCTTCC
UGT2B15	R	AACTGGTCCCACTTCTTCA
SERPINE1	F	ACCCACCGCCGCCTCTTC
SERPINE1	R	CCACCGTGCCACTCTCGT
RABIF	F	GGACCGCTCTCTTCTCTC
RABIF	R	AACTTGATGTTGCCCACG
CENPA	F	TCGTGGTGTGGACTTCAAT
CENPA	R	GCTTCTGCTGCCTCTTGTA
TXNRD1	F	ATAAATGAAAAGACTGGAAAAA
TXNRD1	R	GCCAAAAGTAACTATGGTAAAC
Internal reference H-GAPDH	F	CCCATCACCATCTTCCAGG
Internal reference H-GAPDH	R	CATCACGCCACAGTTTCCC

**Table 2:** Top five differentially expressed adenylate uridylate-rich element genes (DE-AREGs) identified using the multivariate Cox regression analysis.

ID	Coef	HR	HR.95L	HR.95H	*p* value
TXNRD1	0.295306992	1.343538751	1.144714554	1.576896501	0.000301488
CENPA	0.35019523	1.41934462	1.142163909	1.763791637	0.00158281
UGT2B15	0.089964684	0.913963462	0.841185018	0.99303862	0.03358938
SERPINE1	0.080432278	1.08375545	0.977870418	1.201105845	0.125189516
RABIF	0.247936243	1.281378233	0.911795369	1.800766085	0.15326473

**Table 3:** Results of independent prognostic-univariate analysis.

ID	HR	HR.95L	HR.95H	*p* value
Age	1.004326	0.986076	1.022914	0.644558
Gender	0.795005	0.493806	1.279922	0.345073
Grade	1.00493	0.735968	1.372186	0.975314
Stage	1.856679	1.448018	2.380673	0.00000107
T	1.796322	1.42663	2.261814	0.000000629
N	2.007582	0.49043	8.218068	0.332458
M	3.825294	1.199118	12.20303	0.023405
Risk score	1.44637	1.296357	1.613743	0.0000000000395

**Table 4:** Results of independent prognostic-multivariate analysis.

ID	HR	HR.95L	HR.95H	*p* value
T	1.670126	1.313847	2.123019	0.0000279
Risk score	1.392981	1.237027	1.568598	0.0000000447

## Data Availability

TCGA-LIHC dataset was downloaded from TCGA database (https://portal.gdc.cancer.gov/), and GSE14520 and GSE54236 were derived from the GEO database (https://www.ncbi.nlm.nih.gov/gds/). 4884 AREGs were downloaded from the Adenylate Uridylate- (AU-) Rich Element Database (ARED, https://brp.kfshrc.edu.sa/ared).

## Supplementary Materials

Supplementary 1 Figures S1: Gene Ontology (GO) annotation analysis on the downregulated genes (a) and upregulated genes (b).

Supplementary 2 Figures S2: the gene expression levels of the five DE-AREGs in GSE14520 (a) and TCGA (b) datasets.

Supplementary 3 Table S1: the 72 DE-AREGs were selected using the univariate Cox regression analysis.

Supplementary 4 Table S2: the high-risk top 10 of the Gene Ontology (GO) enrichment analysis.

Supplementary 5 Table S3: the low-risk top 10 of the Gene Ontology (GO) enrichment analysis.

Supplementary 6 Table S4: the high-risk top 10 of the Kyoto Encyclopedia of Genes and Genomes (KEGG) enrichment analysis.

Supplementary 7 Table S5: the low-risk top 10 of the Kyoto Encyclopedia of Genes and Genomes (KEGG) enrichment analysis.
