# Supplementary material for: An Inflammation-Associated Prognosis Model for Hepatocellular Carcinoma Based on Adenylate Uridylate- (AU-) Rich Element Genes
Source: Mediators Inflamm. 2023 May 2;2023:2613492. doi: 10.1155/2023/2613492 (PMC10169245; doi:10.1155/2023/2613492)
Supplement: Supplementary 1 — Figures S1: Gene Ontology (GO) annotation analysis on the downregulated genes (a) and upregulated genes (b). [file 2613492.f1.docx]

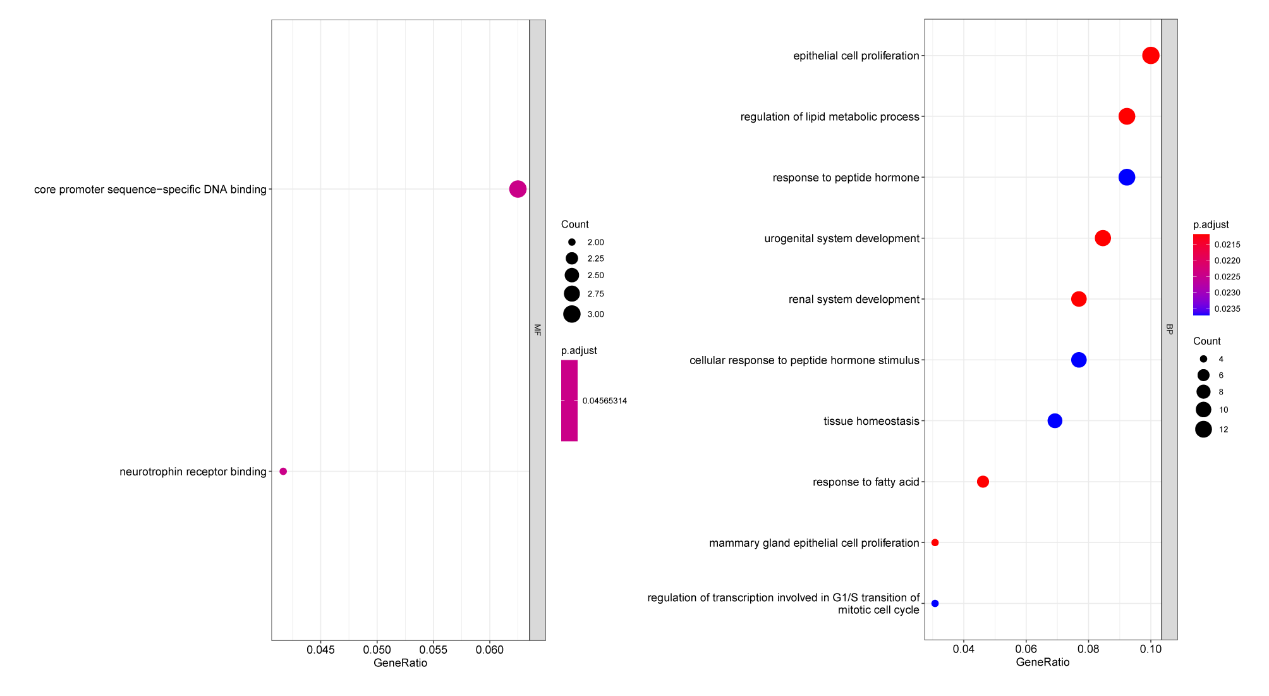


**Figures S1** GO annotation analysis on down-regulated genes (a) and up-regulated genes (b) respectively.
