# Supplementary figures and images for: An Inflammation-Associated Prognosis Model for Hepatocellular Carcinoma Based on Adenylate Uridylate- (AU-) Rich Element Genes
Source: Mediators Inflamm. 2023 May 2;2023:2613492. doi: 10.1155/2023/2613492 (PMC10169245; doi:10.1155/2023/2613492)

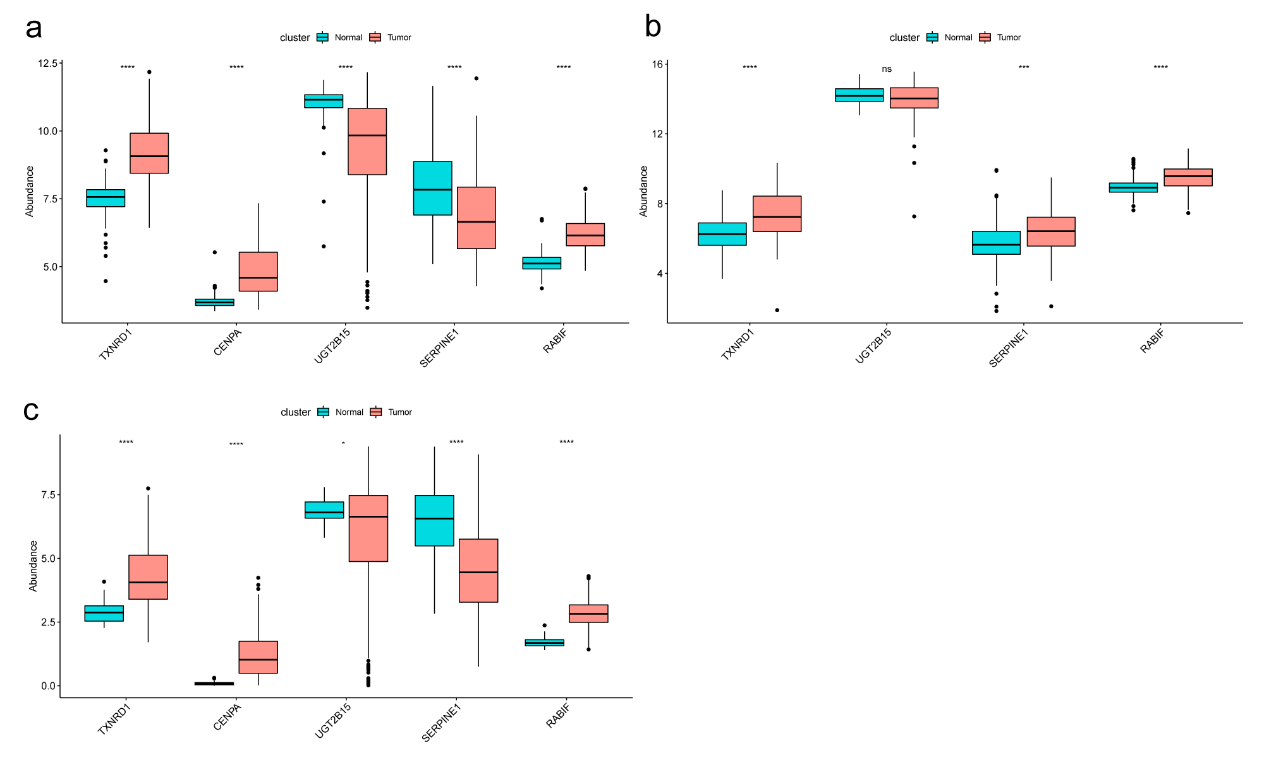


**Figures S2** The gene expression levels of 5 DE-AREGs in GSE14520 (a), GSE54236 (b) and TCGA (c) datasets.

Supplement: Supplementary 2 — Figures S2: the gene expression levels of the five DE-AREGs in GSE14520 (a) and TCGA (b) datasets. [file 2613492.f2.docx]
