# Supplementary material for: An Inflammation-Associated Prognosis Model for Hepatocellular Carcinoma Based on Adenylate Uridylate- (AU-) Rich Element Genes
Source: Mediators Inflamm. 2023 May 2;2023:2613492. doi: 10.1155/2023/2613492 (PMC10169245; doi:10.1155/2023/2613492)
Supplement: Supplementary 3 — Table S1: the 72 DE-AREGs were selected using the univariate Cox regression analysis. [file 2613492.f3.docx]

**Table S1** 72 DE-AREGs selected by univariate cox regression analysis.

| id | HR | HR.95L | HR.95H | pvalue |
| --- | --- | --- | --- | --- |
| CENPA | 1.732506472 | 1.424326707 | 2.107366702 | 3.81E-08 |
| CCT5 | 2.011950634 | 1.553325124 | 2.605987176 | 1.18E-07 |
| NCAPG | 1.671750688 | 1.366633311 | 2.044989201 | 5.80E-07 |
| EZH2 | 1.793818422 | 1.424461961 | 2.258947322 | 6.78E-07 |
| STIP1 | 2.046427594 | 1.536580365 | 2.725445406 | 9.67E-07 |
| CCT6A | 2.110315084 | 1.557575824 | 2.859205752 | 1.44E-06 |
| CBX3 | 2.026200928 | 1.511722316 | 2.715770057 | 2.30E-06 |
| MAFG | 1.798286024 | 1.409037316 | 2.295065281 | 2.41E-06 |
| PBK | 1.485630792 | 1.254049124 | 1.759977984 | 4.69E-06 |
| MCM6 | 1.534839386 | 1.27068346 | 1.853909344 | 8.75E-06 |
| UBE2S | 1.476330557 | 1.240842519 | 1.756509695 | 1.11E-05 |
| LMNB2 | 1.559044942 | 1.278431318 | 1.901252807 | 1.15E-05 |
| ATP1B3 | 1.437206309 | 1.217815528 | 1.696120576 | 1.77E-05 |
| BUB1B | 1.584651709 | 1.283245349 | 1.956851852 | 1.90E-05 |
| TXNRD1 | 1.385515372 | 1.189843777 | 1.613365454 | 2.70E-05 |
| KIF11 | 1.54769909 | 1.254896153 | 1.908821272 | 4.47E-05 |
| NEK2 | 1.422024672 | 1.193368593 | 1.69449253 | 8.27E-05 |
| CDC6 | 1.416956721 | 1.185758499 | 1.693233783 | 0.000125719 |
| ALDH2 | 0.703849555 | 0.587362795 | 0.843438161 | 0.00014208 |
| SLC2A2 | 0.856148356 | 0.788135445 | 0.930030508 | 0.000235477 |
| B4GALT3 | 1.723449613 | 1.272337081 | 2.334506013 | 0.000438896 |
| RABIF | 1.751299651 | 1.279831525 | 2.396448601 | 0.000462053 |
| STK39 | 1.314046693 | 1.12260054 | 1.538141708 | 0.000675229 |
| SCAMP3 | 1.510597138 | 1.187487981 | 1.921622577 | 0.000781056 |
| PRC1 | 1.340940886 | 1.124060212 | 1.599667384 | 0.001117361 |
| DTL | 1.344810342 | 1.119294042 | 1.615763854 | 0.001559733 |
| RPN2 | 1.69757039 | 1.219814056 | 2.362446322 | 0.00169927 |
| NUP210 | 1.403740849 | 1.135088398 | 1.735977899 | 0.001753755 |
| E2F1 | 1.234797943 | 1.079416871 | 1.412545979 | 0.002114219 |
| SHC1 | 1.472869338 | 1.148274196 | 1.889221314 | 0.002300137 |
| NSUN5 | 1.543870875 | 1.167235441 | 2.042036418 | 0.002336515 |
| NPLOC4 | 1.613751527 | 1.175793115 | 2.214840313 | 0.003052125 |
| CTNNA1 | 1.617123659 | 1.174580551 | 2.226402376 | 0.003215648 |
| ENAH | 1.344922282 | 1.103371822 | 1.639353034 | 0.003347456 |
| ATP6V1C1 | 1.450314397 | 1.130991268 | 1.859794951 | 0.003388459 |
| ARPC5 | 1.595362327 | 1.162966824 | 2.18852413 | 0.003779582 |
| GYS2 | 0.868399193 | 0.787876943 | 0.957150942 | 0.004482508 |
| UGT2B15 | 0.900233126 | 0.837102867 | 0.968124365 | 0.004607998 |
| TFRC | 1.332676838 | 1.091950617 | 1.626472413 | 0.00472261 |
| CDK5 | 1.603347513 | 1.154681051 | 2.226349213 | 0.004822161 |
| CPEB3 | 0.629623007 | 0.45554012 | 0.870230993 | 0.005082876 |
| DENND4B | 1.516381152 | 1.128812508 | 2.037018353 | 0.005700172 |
| CYP7A1 | 0.893904087 | 0.824710575 | 0.96890296 | 0.006362547 |
| IPO9 | 1.519234923 | 1.123881282 | 2.053664198 | 0.006540554 |
| UBE2Q1 | 1.509337934 | 1.12013716 | 2.033769686 | 0.006818345 |
| PDK4 | 0.856574457 | 0.764758319 | 0.95941395 | 0.00744648 |
| BHMT | 0.903392619 | 0.838567433 | 0.973229095 | 0.007490322 |
| RAD21 | 1.384617298 | 1.089850635 | 1.759108084 | 0.007711958 |
| RRAGD | 1.307029364 | 1.070437333 | 1.595913842 | 0.00858764 |
| TBC1D16 | 1.416796087 | 1.089084827 | 1.843117361 | 0.00943755 |
| ROBO1 | 1.200122855 | 1.045612145 | 1.377465701 | 0.009479536 |
| PSMD10 | 1.459572361 | 1.096836904 | 1.942268235 | 0.009485865 |
| SLC12A7 | 1.394321543 | 1.08402355 | 1.793441262 | 0.009649634 |
| STEAP4 | 0.693602851 | 0.523385658 | 0.919178634 | 0.010878906 |
| ATXN2L | 1.410728931 | 1.07779174 | 1.846512684 | 0.012231029 |
| DRAP1 | 1.494551993 | 1.091390201 | 2.046642582 | 0.012238743 |
| C7 | 0.886774178 | 0.806798327 | 0.974677831 | 0.012708856 |
| SERPINE1 | 1.129415426 | 1.02401039 | 1.245670178 | 0.014907557 |
| ESR1 | 0.630555711 | 0.431951238 | 0.920475438 | 0.016880593 |
| CBX4 | 1.332805467 | 1.049321164 | 1.692875808 | 0.01854576 |
| GDI1 | 1.425034699 | 1.056137207 | 1.922784161 | 0.020487487 |
| IGF2BP2 | 1.168984291 | 1.021415879 | 1.337872556 | 0.023345858 |
| PCK1 | 0.919674738 | 0.854848095 | 0.989417453 | 0.024753321 |
| LAMC1 | 1.202799859 | 1.022295517 | 1.415175434 | 0.026030437 |
| INPPL1 | 1.372074579 | 1.033800034 | 1.821037519 | 0.028515859 |
| FGB | 0.919597654 | 0.852798187 | 0.991629507 | 0.029374367 |
| SOX9 | 1.140663142 | 1.013093208 | 1.284296837 | 0.029635133 |
| CRHBP | 0.77567024 | 0.615614915 | 0.977338768 | 0.031211907 |
| CTTN | 1.335491573 | 1.018242131 | 1.751585095 | 0.036563805 |
| GNE | 0.836847538 | 0.706882472 | 0.99070755 | 0.038604397 |
| MRPL13 | 1.295065495 | 1.008250723 | 1.663469807 | 0.042939864 |
| DDIT3 | 1.194190385 | 1.003329252 | 1.421358613 | 0.045784807 |
