# Supplementary material for: An Inflammation-Associated Prognosis Model for Hepatocellular Carcinoma Based on Adenylate Uridylate- (AU-) Rich Element Genes
Source: Mediators Inflamm. 2023 May 2;2023:2613492. doi: 10.1155/2023/2613492 (PMC10169245; doi:10.1155/2023/2613492)
Supplement: Supplementary 4 — Table S2: the high-risk top 10 of the Gene Ontology (GO) enrichment analysis. [file 2613492.f4.docx]

**Table S2** high risk top10 of GO significant enrichment.

| NAME | SIZE | ES | NES | NOM p-val | FDR q-val |
| --- | --- | --- | --- | --- | --- |
| GOBP_NUCLEOTIDE_PHOSPHORYLATI ON | 131 | 0.5730612 | 2.121426 | 0 | 0.2222866 |
| GOBP_NEGATIVE_REGULATION_OF_C ELL_CYCLE_PROCESS | 342 | 0.6049341 | 2.1210163 | 0 | 0.1713783 |
| GOBP_NUCLEOTIDE_SUGAR_METABO LIC_PROCESS | 37 | 0.6740771 | 2.1166337 | 0 | 0.1426473 |
| GOBP_NEGATIVE_REGULATION_OF_C ELL_CYCLE_PHASE_TRANSITION | 252 | 0.59903425 | 2.1147685 | 0 | 0.120867 |
| GOBP_NUCLEAR_TRANSPORT | 343 | 0.6222327 | 2.1102939 | 0 | 0.1151303 |
| GOBP_REGULATION_OF_SIGNAL_TRA NSDUCTION_BY_P53_CLASS_MEDIATO R | 176 | 0.6503236 | 2.1064916 | 0 | 0.1041381 |
| GOBP_SIGNAL_TRANSDUCTION_BY_P 53_CLASS_MEDIATOR | 262 | 0.6289818 | 2.1064594 | 0 | 0.0925672 |
| GOBP_MITOTIC_CELL_CYCLE_CHECK POINT | 157 | 0.7102114 | 2.1048477 | 0 | 0.08724535 |
| GOBP_NEGATIVE_REGULATION_OF_MI TOTIC_CELL_CYCLE | 306 | 0.59515357 | 2.1034546 | 0 | 0.0799676 |
| GOBP_NCRNA_METABOLIC_PROCESS | 484 | 0.60223734 | 2.096 | 0 | 0.0810285 |
