# Supplementary material for: An Inflammation-Associated Prognosis Model for Hepatocellular Carcinoma Based on Adenylate Uridylate- (AU-) Rich Element Genes
Source: Mediators Inflamm. 2023 May 2;2023:2613492. doi: 10.1155/2023/2613492 (PMC10169245; doi:10.1155/2023/2613492)
Supplement: Supplementary 5 — Table S3: the low-risk top 10 of the Gene Ontology (GO) enrichment analysis. [file 2613492.f5.docx]

**Table S3** low risk top10 of GO significant enrichment.

| NAME | SIZE | ES | NES | NOM p-val | FDR q-val |
| --- | --- | --- | --- | --- | --- |
| GOBP_MONOCARBOXYLIC_ACID_CAT ABOLIC_PROCESS | 131 | -0.6496406 | -1.967424 | 0.00188679 | 0.1101205 |
| GOBP_BLOOD_COAGULATION_INTRIN SIC_PATHWAY | 17 | -0.8651925 | -1.9614254 | 0 | 0.0884697 |
| GOBP_EPOXYGENASE_P450_PATHWAY | 20 | -0.8661588 | -1.94108 | 0.00192678 | 0.0753883 |
| GOBP_BENZENE_CONTAINING_COMPO UND_METABOLIC_PROCESS | 25 | -0.7318798 | -1.9042867 | 0.001996 | 0.0975799 |
| GOBP_CELLULAR_AMINO_ACID_CATA BOLIC_PROCESS | 105 | -0.67545867 | -1.9005322 | 0.00198807 | 0.0893911 |
| GOBP_ORGANIC_ACID_CATABOLIC_PR OCESS | 257 | -0.5969922 | -1.8868893 | 0.00573613 | 0.0924061 |
| GOBP_FATTY_ACID_CATABOLIC_PROC ESS | 106 | -0.6140139 | -1.8861785 | 0.0056926 | 0.0761865 |
| GOBP_2_OXOGLUTARATE_METABOLIC _PROCESS | 16 | -0.788724 | -1.8785019 | 0 | 0.0697785 |
| GOBP_SHORT_CHAIN_FATTY_ACID_M ETABOLIC_PROCESS | 17 | -0.7947254 | -1.8711804 | 0.00194931 | 0.0704199 |
| GOBP_FATTY_ACID_BETA_OXIDATION | 75 | -0.61913633 | -1.8603978 | 0.0057915 | 0.0695975 |
