# Supplementary material for: An Inflammation-Associated Prognosis Model for Hepatocellular Carcinoma Based on Adenylate Uridylate- (AU-) Rich Element Genes
Source: Mediators Inflamm. 2023 May 2;2023:2613492. doi: 10.1155/2023/2613492 (PMC10169245; doi:10.1155/2023/2613492)
Supplement: Supplementary 6 — Table S4: the high-risk top 10 of the Kyoto Encyclopedia of Genes and Genomes (KEGG) enrichment analysis. [file 2613492.f6.docx]

**Table S4** high risk top10 of KEGG significant enrichment.

| NAME | SIZE | ES | NES | NOM p-val | FDR q-val |
| --- | --- | --- | --- | --- | --- |
| KEGG_PYRIMIDINE_METABOLISM | 98 | 0.6056219 | 2.1155612 | 0 | 0.0551107 |
| KEGG_CELL_CYCLE | 125 | 0.6907384 | 2.0095131 | 0 | 0.0597869 |
| KEGG_LYSOSOME | 121 | 0.55231637 | 1.9902574 | 0 | 0.0479771 |
| KEGG_BLADDER_CANCER | 42 | 0.63961196 | 1.9597331 | 0 | 0.0445429 |
| KEGG_PURINE_METABOLISM | 159 | 0.50793004 | 1.9565382 | 0 | 0.0365121 |
| KEGG_SNARE_INTERACTIONS_IN_VES ICULAR_TRANSPORT | 38 | 0.60831815 | 1.9525374 | 0 | 0.031939 |
| KEGG_UBIQUITIN_MEDIATED_PROTEO LYSIS | 135 | 0.59569633 | 1.9453899 | 0 | 0.0286285 |
| KEGG_RNA_DEGRADATION | 59 | 0.63443094 | 1.9354296 | 0 | 0.0282627 |
| KEGG_BASE_EXCISION_REPAIR | 35 | 0.6730827 | 1.9342638 | 0 | 0.0251224 |
| KEGG_OOCYTE_MEIOSIS | 113 | 0.5449343 | 1.933981 | 0 | 0.0226102 |
