# Supplementary material for: An Inflammation-Associated Prognosis Model for Hepatocellular Carcinoma Based on Adenylate Uridylate- (AU-) Rich Element Genes
Source: Mediators Inflamm. 2023 May 2;2023:2613492. doi: 10.1155/2023/2613492 (PMC10169245; doi:10.1155/2023/2613492)
Supplement: Supplementary 7 — Table S5: the low-risk top 10 of the Kyoto Encyclopedia of Genes and Genomes (KEGG) enrichment analysis. [file 2613492.f7.docx]

**Table S5** low risk top10 of KEGG significant enrichment.

| NAME | SIZE | ES | NES | NOM p-val | FDR q-val |
| --- | --- | --- | --- | --- | --- |
| KEGG_PRIMARY_BILE_ACID_BIOSYNT HESIS | 16 | -0.92635816 | -1.9790643 | 0 | 0.050624 |
| KEGG_FATTY_ACID_METABOLISM | 42 | -0.7947974 | -1.9748943 | 0.00391389 | 0.0266577 |
| KEGG_RETINOL_METABOLISM | 62 | -0.679565 | -1.9095943 | 0.01596806 | 0.034275 |
| KEGG_DRUG_METABOLISM_CYTOCHR OME_P450 | 69 | -0.6578739 | -1.9060464 | 0.01190476 | 0.026533 |
| KEGG_VALINE_LEUCINE_AND_ISOLEU CINE_DEGRADATION | 44 | -0.73589927 | -1.8610425 | 0.01547388 | 0.031505 |
| KEGG_TRYPTOPHAN_METABOLISM | 40 | -0.6324592 | -1.8566123 | 0.00567107 | 0.0273038 |
| KEGG_COMPLEMENT_AND_COAGULA TION_CASCADES | 69 | -0.6803172 | -1.8174951 | 0.02325581 | 0.0344848 |
| KEGG_GLYCINE_SERINE_AND_THREO NINE_METABOLISM | 31 | -0.75511 | -1.7575155 | 0.01871102 | 0.0494094 |
| KEGG_PROPANOATE_METABOLISM | 33 | -0.65650725 | -1.7240576 | 0.0403071 | 0.0567616 |
| KEGG_PPAR_SIGNALING_PATHWAY | 69 | -0.5430415 | -1.6918763 | 0.04435483 | 0.0647721 |
